# Inferring serum proteolytic activity from LC-MS/MS data

**DOI:** 10.1186/1471-2105-13-S5-S7

**Published:** 2012-04-12

**Authors:** Piotr Dittwald, Jerzy Ostrowski, Jakub Karczmarski, Anna Gambin

**Affiliations:** 1 Institute of Informatics, University of Warsaw, Banacha 2, 02-097 Warsaw, Poland; 2 Mossakowski Medical Research Centre PAS, Pawinskiego 5, 02-106 Warsaw, Poland; 3 Department of Oncological Genetics, Maria Skłodowska-Curie Memorial Cancer Center and Institute of Oncology, 02-781 Warsaw, Poland; 4 Department of Gastroenterology, Medical Center for Postgraduate Education, 01-813 Warsaw, Poland; 5 College of Inter-Faculty Individual Studies in Mathematics and Natural Sciences, University of Warsaw, Zwirki i Wigury 93, 02-089 Warsaw. Poland

## Abstract

**Background:**

In this paper we deal with modeling serum proteolysis process from tandem mass spectrometry data. The parameters of peptide degradation process inferred from LC-MS/MS data correspond directly to the activity of specific enzymes present in the serum samples of patients and healthy donors. Our approach integrate the existing knowledge about peptidases' activity stored in MEROPS database with the efficient procedure for estimation the model parameters.

**Results:**

Taking into account the inherent stochasticity of the process, the proteolytic activity is modeled with the use of Chemical Master Equation (CME). Assuming the stationarity of the Markov process we calculate the expected values of digested peptides in the model. The parameters are fitted to minimize the discrepancy between those expected values and the peptide activities observed in the MS data. Constrained optimization problem is solved by Levenberg-Marquadt algorithm.

**Conclusions:**

Our results demonstrates the feasibility and potential of high-level analysis for LC-MS proteomic data. The estimated enzyme activities give insights into the molecular pathology of colorectal cancer. Moreover the developed framework is general and can be applied to study proteolytic activity in different systems.

## Background

### Motivation and related research

Recent advances in high throughput technologies, which evaluate tens of thousands of genes or proteins in a single experiment, are providing new methods for identifying biochemical determinants of the disease process. One of the experimental technologies allowing us to study molecular basis underlying specific disease phenotype is mass spectrometry (MS) [1,2]. Observed large variability in mass spectrometry images of blood samples was attributed to *ex vivo *proteolysis.

Paradoxically, one can take advantage of these findings in cancer diagnostics [3,4] as diagnostic peptides originate after *ex vivo *proteolytic processing of high abundance protein fragments.

As development in hardware and software progresses, we can obtain better and better estimates of peptide concentrations in body fluids, which give many insights into the peptide degradation process. Proteolysis modeled in this paper is the process in which a protein is broken down partially, into peptides, or completely, into amino acids, by proteolytic enzymes present in blood serum. Among proteolytic enzymes two main groups are distinguished. One group includes *exopeptidases *which require a free N-terminal amino group, C-terminal amino group or both and cut the peptide not more than three amino acids from the terminus. Enzymes belonging to the second group are called *endopeptidases *and they tend to cleave away from the end of the peptide.

### Our results

In this paper we present formal mathematical model describing serum proteolysis dynamics. We focus here on the activity of peptide cutting enzymes (peptidases). The model parameters are inferred from liquid chromatography tandem mass spectrometry data (LC-MS/MS).

The dynamical changes in peptide composition caused by proteolytic degradation are described by means of biochemical reactions network. It corresponds to Markov process whose evolution is governed by the system of stochastic differential equations (i.e. Chemical Master Equation).

The current approach significantly extends the exopeptidase activity model presented in [5]. The integration with peptidase database MEROPS (http://merops.sanger.ac.uk) [6,7] allows for modeling the endopeptidase activity as well. Moreover the model parameters inferred from MS data correspond directly to specific proteolytic enzymes present in each sample. On the other hand taking the splitting reaction (coming from endopeptidase cleavage *A *→ *B *+ *C*) into account significantly complicates the mathematical description. There is no analytical solution of the CME as in [5]. Instead we calculate the expected amount of peptides in the stationary state of the process. Those values are compared to the MS readouts for the corresponding peptides. The model parameters are calculated to minimize the discrepancy between expected and observed amount of each peptide.

### Organization of the paper

We start by description of our model presented with the use of so called *cleavage graph*, then we present computational methods to interpret MS data (to fill the graph with appropriate readouts values) and to infer the model parameters. The constrained optimization problem is formulated and solved with Levenberg-Marquadt [8] algorithm. We estimate the convergence and statistical significance of estimation procedure outcomes. Finally, identified active peptidases for both groups of healthy donors and colorectal cancer patients are presented. A preliminary version of this paper was presented at 1st IEEE International Conference on Computational Advances in Bio and medical Sciences (ICCABS 2011). In this extended version we have significantly modified the Results section, by updating the MEROPS data, fixing some errors in scripts for data preprocessing and presenting more statistical analyses.

### Model of proteolysis process

To illustrate the process of peptide degradation we introduce the *cleavage graph*, whose vertices correspond to peptides and proteolytic events. More formally, consider the bipartite digraph, (V∪W,E), where the first set  (*peptide nodes*) corresponds to all subsequences of the peptides considered and the second set of *event nodes * corresponds to all possible proteolytic events.

By proteolytic event we mean the cleavage of a specific substrate at specific site made by a specific peptidase. Hence each event node is labelled by a peptidase, and has one ingoing edge and two outgoing edges (leading to peptide prefix and suffix obtained by cutting the substrate at a single site).

Now we visualize the peptide subsequences as particles placed at peptide nodes of the cleavage graph. The particles are flowing through the edges of the graph according to the Petri net operational semantics, i.e. the transition (event node) consumes one substrate particle, and produces two particles. To assure the stationarity of the system we allow for creation and degradation of particle at any node. We also add the source and the sink in the graph modeling the creation of precursor peptides (e.g. caused by the activity of some endopeptidases, which is not captured by our model) and complete degradation of short peptides. The cleavage graph is constructed for every processed MS sample. The peptide nodes are appropriately filled with mass spectrometry readouts and specific enzymes are assigned to event nodes according to data about real cleavage events (see the next section for details).

**Figure 1:**
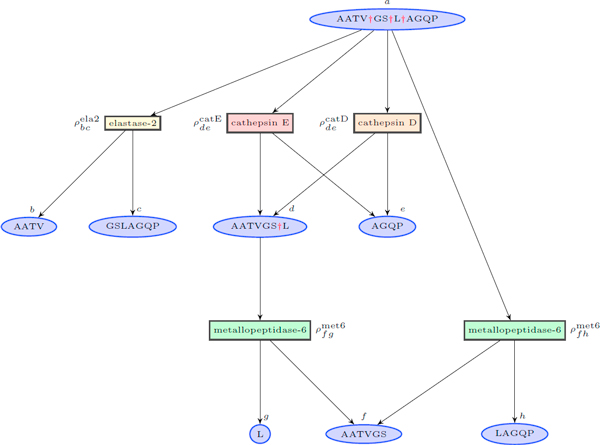
**Exemplary cleavage graph**. The cleavage graph for precursor peptide (*apolipoprotein E *fragment) AATVGSLAGQP, proteolytic events are based on MEROPS database [6].

A small exemplary fragment of the cleavage graph is depicted in Figure 1 five proteolytic events which engage four peptidases are presented. For u,v, w∈V we use the notation u=v†w when peptides  v and  w can be obtained directly by cutting  u ( v is a non-empty strict prefix and *w *is a non-empty strict suffix of  u).

The operation  † can be viewed as string concatenation. To identify a cleavage site we write simply  v†w. Denote by  the set of all peptidases whose activity is modeled. Coefficients $\rho_{vw}^{p}$ (for peptidase p∈P and cleavage v†w) put near the event nodes in Figure 1 correspond to the affinity between the peptidase cleavage pattern and the cleavage site composition (we call them *affinity coefficients*). They are defined for every possible pair of cleavage v†w and peptidase  p and calculated at the graph construction stage. We assume that the cleavage process has reached the equilibrium. Then for every peptide node v∈V the following balance equation [9] holds:

(1) $$\varphi_{v}^{⋆}+\underset{u=v†w}{\sum }x_{u}\rho_{vw}^{p}\lambda_{p}+\underset{u=q†v}{\sum }x_{u}\rho_{qv}^{r}\lambda_{r}=x_{v}\left(\varphi_{v}^{⊥}+\underset{v=x†y}{\sum }\rho_{xy}^{t}\lambda_{t}\right)$$

where $\varphi_{v}^{⋆}$ is an activity of creation the sequence represented by *v*, $\varphi_{v}^{⊥}$ is a degradation activity, $x_{u}$ and $x_{v}$ are expected amounts of peptides  u and  v, $\rho_{xy}^{p}$ is an affinity coefficient and $\lambda_{p}$ is the activity of cleaving by the peptidase  p engaged in the cleavage x†y. Left hand side of the equation above refers to the sum of particles that flow into the node  v, while right hand side to the sum of particles that flow out from this node. From the balance equation above one can easily calculate the expected number of particles in every peptide node $x_{v}$.

## Methods

In this section we describe the process of cleavage graph construction. It has several phases: firstly the set of nodes are determined. Peptide nodes correspond to the sequences identified in tandem MS experiment, while event nodes are selected carefully according to the knowledge from MEROPS database (version 9.4.). During the second stage the graph should be filled with appropriate readouts from LC-MS spectra. To this aim we have to determine which signal in two-dimensional spectral map corresponds to a given peptide sequence (i.e. node in the graph) and to assign to this node the number of particles reflecting the signal strength.

Having the cleavage graph we solve the constrained optimization problem to infer the unknown enzyme activity coefficients which minimize the discrepancy between expected number of peptides (calculated according to the model) and the observed signals in MS samples.

### Cleavage graph construction

Let us define the set of amino acids together with the space letter I={A,C,D,…}∪{-} and the set of loci surrounding (4 from both sides) the cleavage site $J=\left\{P4,\ldots ,P1,P1^{\prime },\ldots ,P4^{\prime }\right\}$.

**Figure 2:**
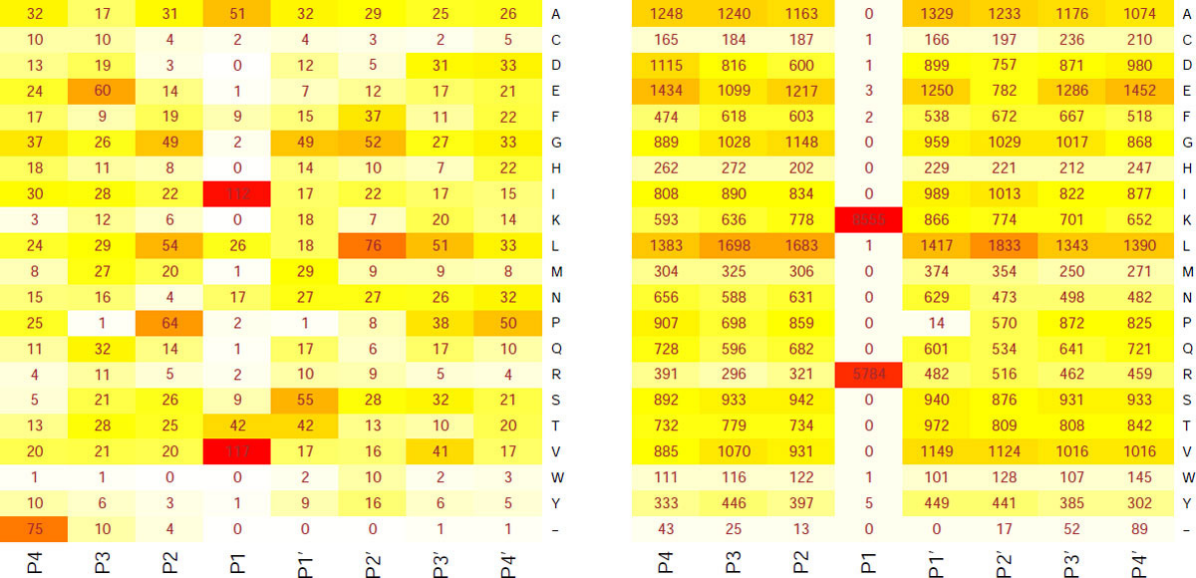
**Frequency matrix for elastase-2 and trypsin-1**. Frequency matrices for elastase-2 (left) and trypsin-1 (right) based on MEROPS database [6]. Color scales are different for each matrix.

For each peptidase p∈P we construct (based on data collected in MEROPS database) the *frequency matrix *$F^{p}={\left(F^{p}\right)}_{ij}$ for i∈I, j∈J. The value $f_{ij}^{p}$ is the frequency of amino acid  i on position  j in all cleavage events, in which *p *is involved. Exemplary frequency matrices for elastase and trypsin enzymes are presented in Figure 2.

Using frequency matrix we construct so called sequence logo [10] to represent the consensus sequence surrounding given cleavage site. Then for any peptidase p∈P we detect the cleavage event cutting given peptide sequence if it matches the consensus sequence well. For more detailed description see Web Supplement (http://bioputer.mimuw.edu.pl/papers/proteolysis/).

#### Affinity coefficients

Let us consider cleavage v†w made by peptidase p∈P. Assume, that the cleavage point is surrounded by the sequence of amino acids (possibly with some empty positions at the ends) $a_{Pk}...a_{P1}a_{P1^{\prime }}...a_{Pl^{\prime }}$ where 1≤k,l≤4.

We define $J^{\prime }=\left\{Pk,\ldots ,P1,\;P1^{\prime },\ldots ,Pl^{\prime }\right\}\subseteq J$. Then we calculate the coefficients $\rho_{vw}^{p}$ as follows ( γ is the normalization constant):

(2) $$\rho_{vw}^{p}=\gamma {\left(\underset{j\in J^{\prime }}{\prod }f_{ja_{j}}^{p}\right)}^{\frac{8}{k+l}}$$

#### Filling the graph with LC-MS readouts

MS samples were acquired from the blood serum of 20 colorectal cancer patients and 19 healthy donors. Each sample was digested by trypsin before LC-MS processing. Having so called *precursor peptides *(which were sequenced by tandem MS), we determine all their subsequences that can be observed during the cleavage process; they form the peptide nodes of the cleavage graph. To this end we digest the precursor peptides *in silico *using the set of human enzymes from MEROPS.

Then we look for corresponding signals in MS spectra as follows: using mz2m tool [11] we obtain a list of mono-isotopic peak coordinates (*m/z*, retention time and charge) together with their intensities. The search is processed for each sequence charged by each of eight possible charges (values from 1 to 8) detected by FTICR spectrometer. Expected value of retention time for each peptide is predicted from its amino acid composition by linear regression model [12] (trained by the set of known retention times for precursor peptides). Then we look for MS signals that are closest to expected ones (nearest neighbor classifier) and their distances do not exceed given threshold. Notice, that we ignore LC-MS signals corresponding to peptides not sequenced by tandem MS and theirs degraded forms. Therefore we use only partial information about degradation scheme, and possibly the activity of some enzymes engaged in the process cannot be inferred. By applying this procedure we fill many peptide nodes by appropriate peptide amount. However a large percent of the nodes remain empty. Finally, we prune the cleavage graph by removing recursively empty sources and empty leaves.

### Constrained optimization

Let us denote by , L⊂V respectively, sets of sources (i.e. nodes without ingoing edges) and leaves (nodes without outgoing edges) in the cleavage graph. Let n=|S|+|L|+|P| and $z=\left({\left(\varphi_{u}^{⋆}\right)}_{u\in S},{\left(\varphi_{w}^{⊥}\right)}_{w\in L},{\left(\lambda_{p}\right)}_{p\in P}\right)\in R^{n}$ denote the vector of model parameters to be inferred. We are mainly interested in estimation of the parameters ${\left(\lambda_{p}\right)}_{p\in P}$ which describes activities of peptidases. We define $\Phi ={\left(\phi_{v}\right)}_{v\in V}$ recursively for all v∈V sorted topologically:

1. if v∈S then $\phi_{v}\left(z\right)=\frac{\varphi_{v}^{⋆}}{\underset{v=x†y}{\sum }\rho_{xy}^{t}\lambda_{t}}$,

2. if v∉S and v∉L then $\phi_{v}\left(z\right)=\frac{\underset{u=v†w}{\sum }\phi_{u}\left(z\right)\rho_{vw}^{p}\lambda_{p}+\underset{u=q†v}{\sum }\phi_{u}\left(z\right)\rho_{qv}^{r}\lambda_{r}}{\underset{v=x†y}{\sum }\rho_{xy}^{t}\lambda_{t}}$,

3. if v∉L then $\phi_{v}\left(z\right)=\frac{\underset{u=v†w}{\sum }\phi_{u}\left(z\right)\rho_{vw}^{p}\lambda_{p}+\underset{u=q†v}{\sum }\phi_{u}\left(z\right)\rho_{qv}^{r}\lambda_{r}}{\varphi_{v}^{⊥}}$.

The graph pruning grants that S∩L=0̸, and the topological ordering assures that the function  Φ is well-defined.

Denote by *y_i _*the amount of peptide sequences identified in LC-MS/MS experiment in the *i*-th peptide node, and by O⊆V set of vertices with $y_{i}>0,|O|\;=m$. We solve non-linear least squares problem with objective function $\Psi ={\left(\psi_{v}\right)}_{v\in O}$ defined for each v∈O by the formula $\psi_{v}\left(z\right)=\phi_{v}\left(z\right)-y_{v}$, which is well formulated and solvable for m≥n (fortunately holding in our case for all investigated MS samples). We applied Levenberg-Marquadt algorithm (LMA) [13] to find optimal configuration of model parameters.

### Compositional data

To make the outcome of estimation procedure comparable across different MS samples we normalized the vector of parameters corresponding to peptidases' activities. Notice, that normalization does not change the value of function *ϕ_v _*for any v∈O. The normalized activities, say vector  x, lies on the simplex, therefore we should apply the appropriate transformation (called centered log ratio clr(x)) to deal with them in Euclidean space (see the theory of compositional data analysis [14] for details). Let g(x) denotes the geometric mean for vector  x, centered log ratio is defined as follows:

$$clr\left(x\right)={\left(\text{ln}\frac{x_{i}}{g\left(x\right)}\right)}_{i=1,\ldots ,m}$$

## Results

The optimization procedure was applied to infer the enzymatic activity for 39 LC-MS samples, i.e. for each sample we obtained optimal parameters $ẑ=\left({\left({\hat{\varphi }}_{u}^{⋆}\right)}_{u\in S},{\left({\hat{\varphi }}_{w}^{⊥}\right)}_{w\in L},{\left({\hat{\lambda }}_{p}\right)}_{p\in P}\right)$.

We run LMA for each data set 7 times (each time from different starting point) and use the maximal number of iterations set up to 200 as a stop criterion. To measure the quality of estimation we use relative squared errors (rse) [15], i.e.:

$$\text{rse}\left(ẑ\right)=\frac{\sum_{i\in O}{\left({ŷ}_{i}-y_{i}\right)}^{2}}{\sum_{i\in O}{\left({ȳ}_{i}-y_{i}\right)}^{2}}$$

where ${ŷ}_{i}=\phi_{i}\left(ẑ\right)$ and ${ȳ}_{i}=\frac{1}{m}\sum_{i\in O}y_{i}$.

### Adequacy of the model

**Figure 3:**
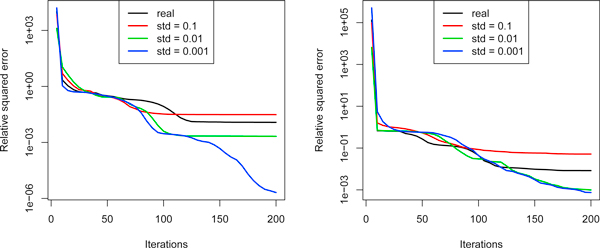
**Comparison of median value of relative squared error**. Comparison of median value of relative squared errors for real data and synthetic data generated according to the model (plot for the sample no. 5 on the left and for the sample no. 19 on the right).

Aiming in justifying the adequacy of the proposed model we made the following experiment. The estimation procedure was run to obtain the expected number of peptide sequences ${ŷ}_{v}$ in every peptide node v∈O. Then we have filled the cleavage graph with synthetic data $y_{v}^{⋆}$ generated independently from normal distributions with mean ${ŷ}_{v}$ and standard deviation *σ *∈ {0.1, 0.01, 0.001} (with additional constraint $y_{v}^{⋆}>0$). In this way we obtained three synthetic data sets, which are in the good, moderate and weak accordance with our model. As we expect, the quality of estimation procedure reflects the discrepancy with the model. Figure 3 compares median relative squared error for real data and three data sets generated from the model with different level of discrepancy.

### Statistical significance of estimation quality

Optimization procedure yielded rather small rse errors for most samples. However, we were interested how the final relative squared error depends on the input data, and whether results obtained by us are statistically significant. To answer this question for each MS sample  x we have generated vector *ζ *of 1000 randomly permuted variants (i.e. the topology of cleavage graph remained the same, while the amounts of peptides assigned to nodes were permuted). Then we run the optimization procedure for all data set to obtain the experimental distribution of rse values. Now, we can define *p-value *for rse(x) as follows:

(3) $$\text{p-val(rse(}x\text{))}=\frac{\left|\{i:\text{rse(}\zeta_{i}\text{)}\leq \text{rse(}x\text{)\}}\right|}{1000}$$

Table 1 presents *p-values *and corresponding rse values for all analysed MS samples.

**Table 1:** Final relative squared errors (rse) and p-values (calculated from rse distribution).

sample	final rse	p-value	sample	final rse	p-value
19	0.008	*<*0.001	20	0.011	*<*0.001
5	0.012	*<*0.001	1	0.016	0.001
9	0.034	0.001	2	0.026	0.005
14	0.091	0.005	13	0.061	0.007
4	0.063	0.008	10	0.065	0.008
11	0.031	0.01	30	0.136	0.021
6	0.125	0.026	29	0.163	0.029
15	0.058	0.032	28	0.185	0.057
7	0.131	0.076	24	0.11	0.078
16	0.134	0.093	32	0.392	0.11
8	0.156	0.144	23	0.379	0.215
33	0.45	0.23	22	0.358	0.234
27	0.483	0.257	26	0.471	0.29
21	0.367	0.301	18	0.262	0.317
25	0.521	0.324	12	0.332	0.434
34	0.589	0.436	38	0.589	0.44
35	0.628	0.452	31	0.436	0.623
37	0.478	0.529	36	0.567	0.64
3	0.637	0.729			

### Biological significance of inferred enzymes

**Figure 4:**
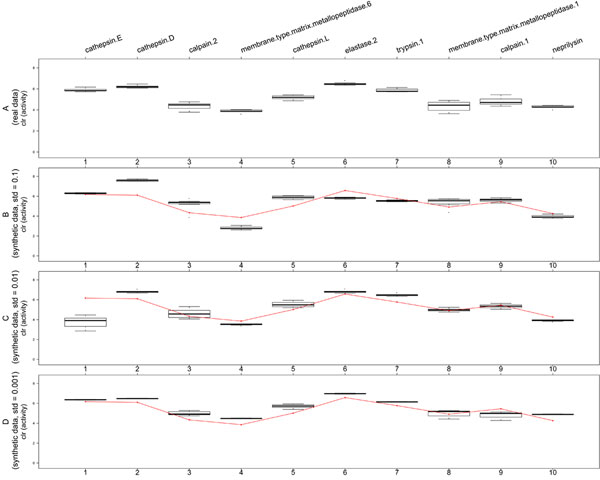
**Peptidases' activities for sample no. 5**. (A) Inferred peptidases' activities for sample no. 5 (healthy donor). (B-D) Same parameters for synthetic data generated from the model with standard deviation set to 0.1, 0.01, 0.001, respectively. Red lines correspond to model peptidases' activities, which we aim to recover.

**Figure 5:**
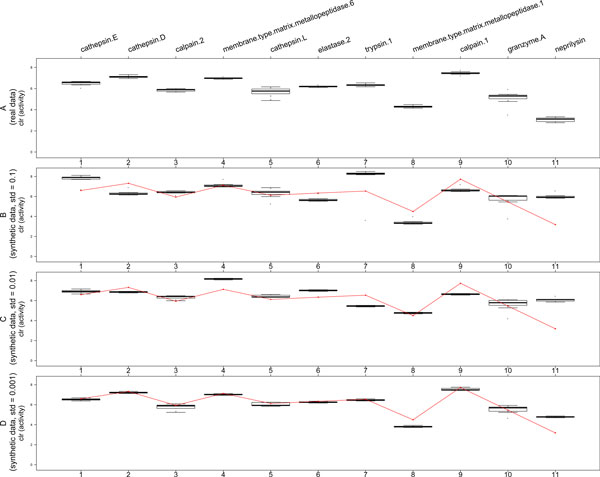
**Peptidases' activities for sample no. 19**. (A) Inferred peptidases' activities for sample no. 19 (colorectal cancer patient). (B-D) Same parameters for synthetic data generated from the model with standard deviation set to 0.1, 0.01, 0.001, respectively. Red lines correspond to model peptidases' activities, which we aim to recover.

Figure 4A and Figure 5A present the inferred peptidases' activities for samples no. 5 (healthy donor) and 19 (colorectal cancer patient). Subfigures B-D illustrate the accuracy of estimation procedure for synthetic data sets for which the estimated parameters are known and equal to those inferred from real data (red line). We observe once more, that the quality of estimation correlates well with the discrepancy of data (for smallest standard deviation, i.e. Figure 4D and Figure 5D, the estimated parameters are close to real ones). Analogous results are obtained for other analyzed samples.

**Figure 6:**
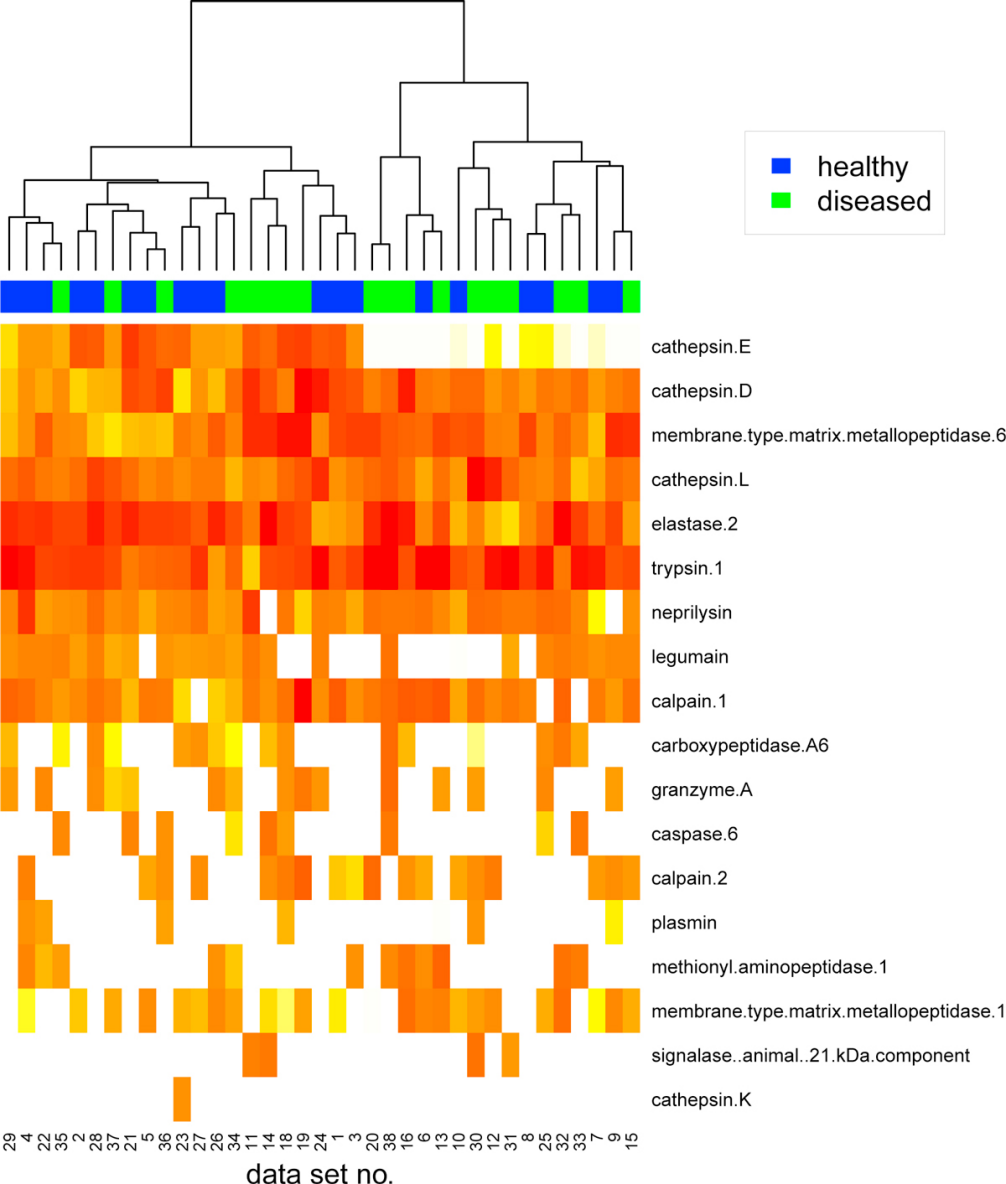
**Peptidases' activities**. Peptidases' activities (after clr transformation) for all analyzed samples. The red-white scale represents peptidase activities in descending order.

The set of identified enzymes do not vary significantly between all investigated samples: there are 6 peptidases identified in all samples and 19 peptidases found in at least one sample (listed in Figure 6). For further analysis we selected 37 samples (19 healthy, 18 diseased), for which acceptable estimates had been obtained (c.f. Table 1). We excluded two colorectal cancer samples (no. 17 and 39) as they perform significantly different than others (one required much higher threshold in nearest neighbor classifier during MS signal detecting phase and the other returned relative squared error much higher than 1).

Heatmap in Figure 6 presents the activities of peptidases identified for these samples. Hierarchical clustering of activity profiles groups samples into clusters being in good accordance with patient's diagnosis. The diverse serum proteolytic activity for cancer patients and healthy donors has been reported in many papers (see e.g. [3]). In [16] has been showed that patients exhibit different enzymatic activities than healthy subjects for following peptidases (identified by our method as well): trypsin, cathepsin D and elastase (c.f. Figure 6). We have also detected the family of matrix metallopeptidases, whose role in cancer development and progression is significant [17,18]. Similarly calpain enzyme is used as a marker for the early detection of colorectal carcinoma [19] and inhibitors of cathepsins as possible therapeutics in colorectal diseases [20]. Moreover, cathepsins (because of their ability to degrade extracellular matrix proteins) have been implicated to play a role in invasion and metastasis of colorectal cancer.

**Figure 7:**
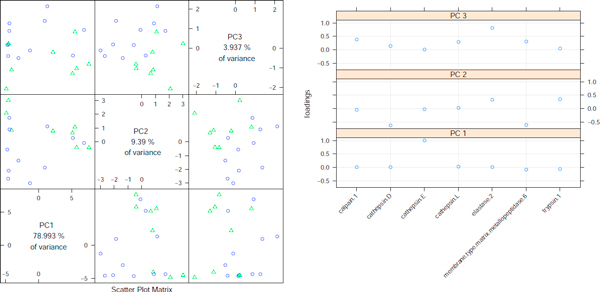
**Principal component analysis**. Principal component analysis scatterplot for 19 samples with best p-values. Corresponding loadings on the left panel.

We have conducted principal component analysis for enzyme activities inferred for 19 samples having smallest p-values (c.f.Table 1). The scatterplots in Figure 7 illustrate the outcome of the analysis. Well separation of two groups of patients is visible on projection to the plane determined by the second and third principal component. Closer look at corresponding loadings suggests the crucial role of elastase and cathepsin enzymes in those components.

## Conclusions

In this paper we significantly extend formal model of protein degradation proposed in [5]. The extension is twofold: firstly current approach encompasses endopeptidase activity as well (while [5] deals with exopeptidases only), and secondly we integrate our model with knowledge about proteolytic events stored in MEROPS database [6]. Moreover, we formulate the task of inferring parameters of our model as constrained optimization problem, which we solve by standard procedure for non-linear least squares. This approach turned out to be more time efficient for complex MS data when comparing to previous Markov Chain Monte Carlo method (in [5] the Metropolis-Hastings algorithm was applied to sample parameters from the posterior distribution).

Being aware of the problems with quality and reproducibility of the LC-MS experiments we selected for detailed analysis only a part of accessible data, namely those for which the parameter estimation procedure converges and yields small error. The expected retention time for investigated substances is obtained by rather unsophisticated approach (i.e. linear regression model), which may have impact on the analysis. Preliminary outcomes for these samples are very promising: identified enzymes are known to play a crucial role in colorectal cancer. However, our results are far from any medical diagnosis. The proposed method constitutes the proof of concept and requires more profound investigations meeting all clinical standards. We also should discuss here the limitations of our methods applied to MS data obtained by present technologies. There is a lot of tryptic peptides which are not identified by tandem mass spectrometry. LC-MS signals corresponding to these peptides and theirs degraded forms are missed during cleavage graph filling phase. Therefore the inference of proteolytic enzymes' activities is based on only partial information and could be incomplete as well. However, it is worth to noting here that our method would demonstrate its full potential while applied to high quality data hopefully obtained from the future MS technologies. One direction for further development is to focus on cleavage detection and to apply recently proposed *ice-logo *instead of sequence logo [21]. Ice-logo contains the information not only about residues that are statistically overrepresented but also about those, that are underrepresented. This approach could be valuable especially in cases, when, as in the case of data stored in MEROPS database, we know only fraction of all proteolytic events.

## Competing interests

The authors declare that they have no competing interests.

## Authors' contributions

AG developed strategy for the study, and prepared the final version of the manuscript. PD implemented algorithms for estimation of enzymatic activity and participated in drafting the manuscript. JO and JK provided the LC-MS/MS samples and participated in the design of the study. All authors read and approved the final manuscript.
